# Copy Number and Loss of Heterozygosity Detected by SNP Array of Formalin-Fixed Tissues Using Whole-Genome Amplification

**DOI:** 10.1371/journal.pone.0024503

**Published:** 2011-09-26

**Authors:** Angela Stokes, Ignat Drozdov, Eliete Guerra, Christos A. Ouzounis, Saman Warnakulasuriya, Michael J. Gleeson, Mark McGurk, Mahvash Tavassoli, Edward W. Odell

**Affiliations:** 1 Department of Oral Pathology, King's College London Dental Institute, London, United Kingdom; 2 Cardiovascular Division, British Heart Foundation Centre of Research Excellence, King's College London, London, United Kingdom; 3 Department of Informatics, Centre for Bioinformatics, School of Natural and Mathematical Sciences, King's College London, London, United Kingdom; 4 Oral Pathology, Department of Dentistry, Faculty of Health Science, University of Brasilia, Brasilia, Brazil; 5 Department of Oral Medicine, King's College London Dental Institute, London, United Kingdom; 6 Department of Otolaryngology, Guy's Hospital, London, United Kingdom; 7 Department of Oral and Maxillofacial Surgery, King's College London Dental Institute, London, United Kingdom; Howard University, United States of America

## Abstract

The requirement for large amounts of good quality DNA for whole-genome applications prohibits their use for small, laser capture micro-dissected (LCM), and/or rare clinical samples, which are also often formalin-fixed and paraffin-embedded (FFPE). Whole-genome amplification of DNA from these samples could, potentially, overcome these limitations. However, little is known about the artefacts introduced by amplification of FFPE-derived DNA with regard to genotyping, and subsequent copy number and loss of heterozygosity (LOH) analyses. Using a ligation adaptor amplification method, we present data from a total of 22 Affymetrix SNP 6.0 experiments, using matched paired amplified and non-amplified DNA from 10 LCM FFPE normal and dysplastic oral epithelial tissues, and an internal method control. An average of 76.5% of SNPs were called in both matched amplified and non-amplified DNA samples, and concordance was a promising 82.4%. Paired analysis for copy number, LOH, and both combined, showed that copy number changes were reduced in amplified DNA, but were 99.5% concordant when detected, amplifications were the changes most likely to be ‘missed’, only 30% of non-amplified LOH changes were identified in amplified pairs, and when copy number and LOH are combined ∼50% of gene changes detected in the unamplified DNA were also detected in the amplified DNA and within these changes, 86.5% were concordant for both copy number and LOH status. However, there are also changes introduced as ∼20% of changes in the amplified DNA are not detected in the non-amplified DNA. An integrative network biology approach revealed that changes in amplified DNA of dysplastic oral epithelium localize to topologically critical regions of the human protein-protein interaction network, suggesting their functional implication in the pathobiology of this disease. Taken together, our results support the use of amplification of FFPE-derived DNA, provided sufficient samples are used to increase power and compensate for increased error rates.

## Introduction

High-density genotyping SNP arrays provide an opportunity to assess the whole genome for multiple effects of genetic instability, such as copy number changes and loss of heterozygosity (LOH) in the same DNA sample (Reviewed by Dutt *et al.* [1]). Unfortunately, their requirement for relatively large amounts of minimally fragmented DNA is considered to prohibit the investigation of very small samples [2], such as when laser capture micro-dissection (LCM) [2] is required to ensure a homogeneous sample. Samples from formalin-fixed paraffin embedded (FFPE) material suffer further problems of reduced DNA extraction efficiency, and reduced DNA quality due to cross-linking and degradation [3], [4]. The use of whole-genome amplification (WGA) offers the potential to overcome these limitations and for some diseases and tissue types may be the only way to examine limited clinical samples [5].

Ideally, WGA would amplify DNA without errors, but early PCR-based methods using random or degenerate primers were subject to amplification artefacts, incomplete coverage, and low yield (reviewed by Hughes *et al*, [5]). Isothermal amplification methods such as multiple-displacement amplification (MDA) [6] use random primers and a DNA polymerase such as phi29, that generates large DNA fragments and has a reduced error rate compared to Taq polymerase [2]. This is now generally considered to be the WGA method of choice. However, fragmented DNA, such as that from FFPE tissues, does not amplify well with MDA methods because they require high molecular weight sample DNA [7].

An alternative method for fragmented DNA uses a ligation adaptor method (such as OmniPlex®) to increase the likelihood of complete genome coverage [8]. The DNA is initially randomly fragmented, to average size of 400 bp, and universal adaptors are ligated to both ends. Adaptor-specific primers and a high-fidelity polymerase then amplify the fragments by PCR [7], [8], [9]. This method has been shown to be comparable to MDA in terms of genotyping accuracy for unfragmented DNA [9]. Also, because of its ability to work with small DNA fragments, it is suggested to be an appropriate method for degraded DNA such as that from FFPE tissues [8], where fragment sizes are usually within the 300–500 bp range [3].

In this study, we investigated the use of Omniplex® technology to amplify DNA extracted from LCM isolated FFPE tissues from both dysplastic oral epithelium and normal muscle controls, and compared their subsequent performance on Affymetrix Genome-wide Human SNP array 6.0 with that of matched-paired un-amplified gDNA. Furthermore, we provide a systems-wide overview of aberrations in dysplastic oral epithelium by integrating genotype information with high confidence human protein-protein interaction (PPI) network and known cancer phenotypes, using graph theoretic approaches to show how such data can be valid for functional analyses.

## Results

Normal muscle and at least one dysplastic epithelial sample were isolated by laser capture micro-dissection (LCM) from eighteen biopsy samples from six individuals. These tissues ranged from 3–14 years of storage since processing. At least 1.5 cm^2^ of tissue (from 12 µm thick sections) was required to reliably extract 1 µg DNA from most FFPE tissues. Eight of the tissue samples did not produce this quantity of DNA due to small sample size. The ten remaining samples (from four patients) were amplified using OmniPlex® whole-genome amplification. This procedure amplified 0.1 µg of DNA to give an average final yield of 2.1 µg (range 0.6–3.2 µg). All samples, both unamplified and amplified, were able to produce PCR products of at least 350 bp in length. Ten unamplified genomic DNA samples (gDNA) and ten matched WGA amplified samples (WGA DNA) were hybridised to Affymetrix SNP 6.0 arrays (Table S1).

### SNP Call Rate and Concordance

Internal method control DNA, extracted from blood, gave mean SNP call rates of 90.7%±0.5. Before filtering the SNPs to take into account fragment length, the call rates generated by FFPE gDNA and WGA DNA were on average 90.1%±1.2 and 89.8%±0.7 respectively. This was reduced by 6–7%, to 84.1±2.2 and 82.8±1.6, when a 350 bp fragment length filter described in the Affymetrix Technical Note [10] and by Jacobs *et al*, 2007 [4] was applied (see **Table 1**). This 350 bp fragment length filter reduces the SNP repertoire from 909,622 to 131,429.

**Table 1:** SNP Call Rates as a percentage of the 131,429 SNPs remained after a 350 bp filter (n = 10).

Call Rates(%)	Genomic DNA			Amplified DNA		
	All	Homozygous	Heterozygous	All	Homozygous	Heterozygous
**Range**	81.1–87.1	49.2–65.1	22.0–32.2	79.4–84.5	43.6–62.3	21.8–35.8
**Mean**	84.1	56.5	27.5	82.8	54.4	28.4
**St Dev**	2.2	5.5	3.4	1.6	5.4	3.9

WGA DNAs produced slightly lower call rates (1.3% lower) than their gDNA paired counterpart, but the homozygous and heterozygous call rates were similar between the two groups (see **Table 1**). Overall, the homozygous and heterozygous call rates for FFPE and WGA samples were both only slightly lower than that for unfragmented control DNA, which were 62.2±0.5 and 28.5±0.1 respectively.

The SNPs that failed in the WGA samples (no call) were compared with the gDNA, to identify allelic amplification bias. Over half (56.1%±5.0) of the SNPs that failed to give a genotype call in WGA samples, also failed to give a genotype call in the gDNA matched pair. Of those that did give genotype calls in the gDNA matched pair, there was no significant difference in the proportion of allele call rates for AB and BB calls. Yet, AA calls showed a significant 2.23% decrease on average in call rate (paired t test, p = 0.038, see **Table 2**).

**Table 2:** Genotype Distribution of the gDNA overall and the ‘No Calls’ in the amplified DNA (n = 10).

Call Rates(%)	Genotypes in gDNA			Corresponding gDNA genotype of the No Call SNPs in amplified DNA		
	AA	AB	BB	AA	AB	BB
**Range**	30.5–37.9	26.6–39.5	29.9–36.8	27.1–35.8	25.0–43.9	28.6–38.3
**Mean**	33.9	32.9	33.2	31.7	35.6	32.7
**St Dev**	2.5	4.8	2.4	3.4	7.1	3.8

Of the 131,429 SNPs available for detection, a mean of 76.5% were called in both the gDNA and WGA matched samples. Of the SNPs called in both samples, 82.4%±5.9 on average were concordant between the two samples (see **Table 3**). For control DNA samples, concordance with matched WGA DNA was higher at 95.3%. The fragment filter for the control DNA was set at 400 bp because larger DNA fragments were amplifiable from this sample when compared to the FFPE samples, reflecting the improved quality of DNA in these samples before the fragmentation step of the amplification procedure.

**Table 3:** SNP call concordance rates (n = 10).

	SNPs Called in both samples	Concordant SNPs	% Concordance
**Range**	93905–105786	69135–95057	73.6–90.6
**Mean**	100599.2	83120.7	82.4
**St Dev**	3732.2	8908.1	5.9

### Paired Copy Number

Paired copy number analysis was performed on DNA from dysplastic tissues with the Partek Genomics Suite, using SNP 6.0 intensity data (Affymetrix ‘.cel’ files) with the DNA of normal tissues used as a reference. Differences were analysed between the paired samples based on the gene involved and the base pair start and end of the region of change. Changes were described as either amplification (if the average intensity value was over the diploid value of 2) or deletion (if under 2). As expected, the number of copy number changes detected varied greatly between the different dysplastic samples (gDNA range - 1704 to 6421, **Table 4**) reflecting the known genomic instability in the tissues.

**Table 4:** Paired copy number analysis - The number of gene changes and their concordance (n = 6).

	Number of Changes			Concordant Changes		‘missed’ changes% of gDNA pair		‘extra’ changes% of WGA pair	
	gDNA	WGA	in both	Number	%	Amplification	Deletion	Amplification	Deletion
**Range**	1704–6429	1814–4897	836–3244	836–3220	98.8–100.0	2.3–69.3	5.0–48.7	1.71–26.17	10.4–52.2
**Mean**	4076.5	2777.8	1765.8	1755.3	99.5	34.9	15.4	10.2	26.2
**St Dev**	2047.0	1195.9	811.9	804.0	0.4	27.7	16.5	9.0	13.8

Analysis of WGA samples was repeated using WGA normal tissue DNA as a control. The number of changes also varied (range 1814 to 4897) and, in all but one, was less than the matching gDNA pair (not shown). A mean of 99.5%±0.4 of the changes detected in both samples were concordant (**Table 4**). Non-concordant changes (‘mismatch’ changes – amplified in either the gDNA or amplified sample and deleted in the other) were very infrequent in these samples, representing less than 1% of the total changes in either pair.

However, over half of the changes detected in the gDNA pair (mean 50.25±22.08) were not found in the WGA pair. These ‘missed’ changes were mostly amplifications (mean 34.9%±27.7) rather than deletions (mean 15.4%±16.5) when calculated as a percentage of all gDNA changes (**Table 4**). In all but one sample pair, there were more ‘missed’ amplifications than deletions in the WGA samples. There were also a large number of changes detected in the WGA pair that were not found in the gDNA pair (mean 36.4±12.6). These ‘extra’ changes were usually deletions (mean 26.2%±13.8) rather than amplifications (mean 10.2%±9.0). In all but two sample pairs there were more ‘extra’ deletions than amplifications in the WGA samples.

### Paired LOH

Paired LOH analysis was performed on DNA from dysplastic tissue, with the Partek Genomics Suite using genotyping data generated by Affymetrix Birdseed (v2) RLMM algorithm (Affymetrix ‘.chp’ files) using the DNA of normal tissues as reference. LOH was detected when a heterozygous SNP in the reference (normal) sample was detected as homozygous in the test (dysplastic) sample. Changed regions were analysed between the paired samples based on the genes involved in the region of change. The number of LOH changes detected varied greatly between the different dysplastic samples, as expected (gDNA range 77 to 15,446, **Table 5**).

**Table 5:** Paired LOH analysis - The number of gene changes and their concordance (n = 6).

	Number of Changes			Concordant Changes		‘missed’ LOH	‘extra’ LOH
	gDNA	WGA	In both	Number	%	% of gDNA pair	% of WGA pair
**Range**	77–15446	215–12624	15–10436	15–10436	100	32.4–96.0	17.3–97.5
**Mean**	3258.5	2725.8	1828.2	1828.2	100	73.2	74.5
**St Dev**	6018.2	4861.8	4217.9	4219.9	0	22.2	29.0

Analyses of WGA samples were repeated using a WGA normal tissue DNA as a control. The number of LOH changes also varied (range 215 to 12624) and showed no trend towards either an increased or decreased number when compared to the matching gDNA pair (**Table 5**). The majority of the LOH changes detected in gDNA pair were not detected in the WGA pair (‘missed’ changes mean 73.20%+/−22.15) and a similar number (mean 74.49%+/−28.95) of LOH changes in the amplified pair were not found in the gDNA pair (‘extra’ LOH changes – see **Table 5**).

### Copy number and LOH Overlap

When the copy number and LOH analyses were overlapped, the procedure generated data on presence of LOH during amplifications and deletion, and also on copy neutral LOH (acquired uniparental disomy) events. Once again, the number of changes varied greatly between the dysplastic samples, and an increased number of changes were consistently detected in the gDNA pairs, when compared to the amplified pairs (mean values of 7426.7±4710.3 and 5353.0±4485.5 respectively, **Table 6**).

**Table 6:** Copy number and LOH overlap - The number of gene changes their concordance (n = 6).

	Number of Changes			Concordant Changes		‘missed’ Overlap	‘extra’ Overlap
	gDNA	WGA	In both	Number	%	% of gDNA pair	% of WGA pair
**Range**	3857–16148	2490–13995	1553–12163	1435–9819	71.0–93.7	24.7–60.6	9.9–29.5
**Mean**	7426.7	5353.0	4380.8	3693.7	86.5	46.3	20.9
**St Dev**	4710.3	4485.5	3997.9	3194.9	8.9	16.2	8.0

There was a mean of 4380.8±3997.0 changes detected in both gDNA and amplified pairs, and a mean of 86.5%±8.9 of these changes were concordant. Almost 50% of the changes detected in the gDNA pairs were not found (‘missed’ changes) in the WGA samples (46.3%±16.2 – **Table 6** **, Figure S1**), and a mean of 20.9%±8.0 of changes in the amplified pairs were not detected in the gDNA pairs (‘extra’ changes, **Figure S1**).

### Chromosomal Location of ‘missed’ and ‘extra’ changes

**Figure 1:**
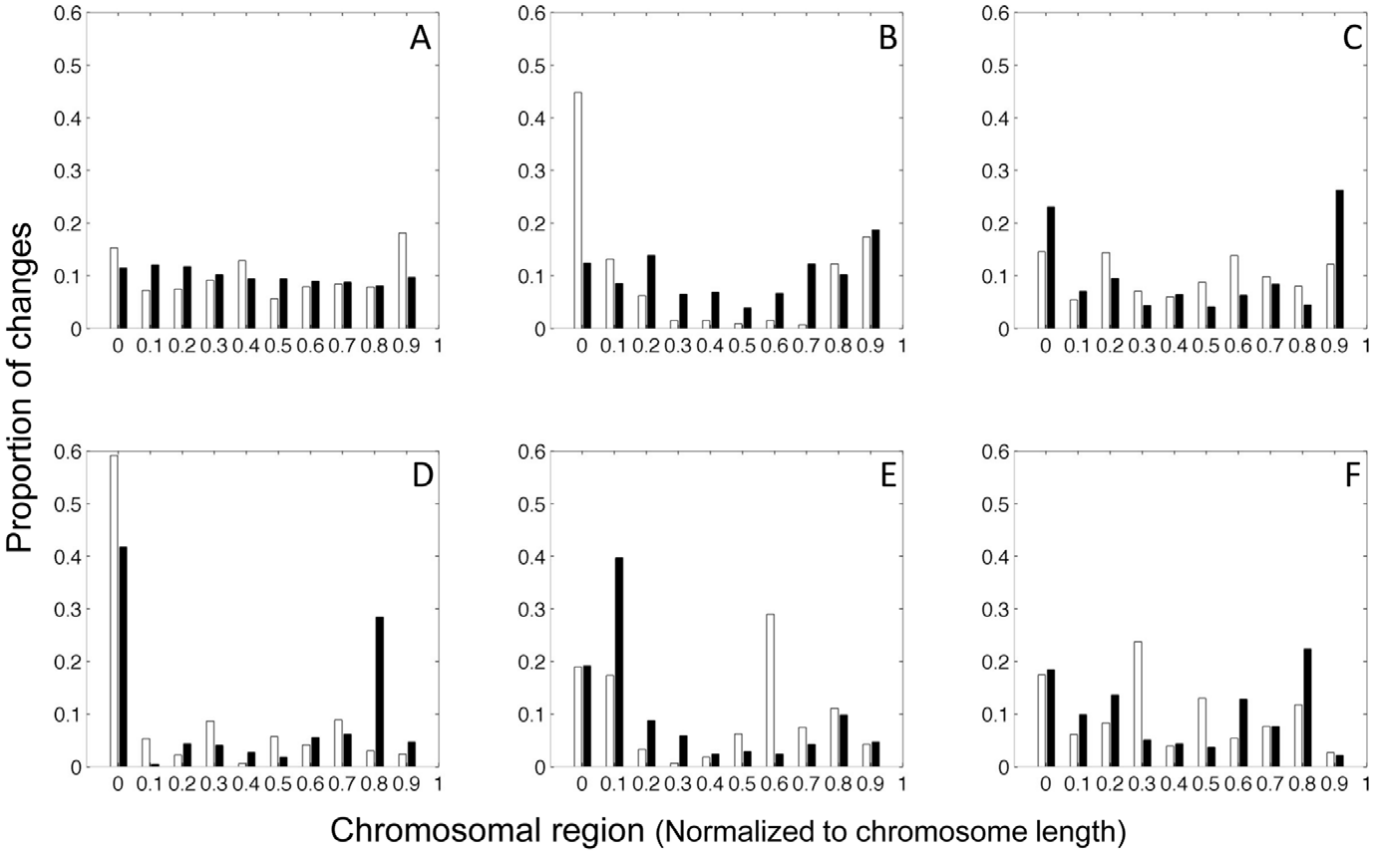
Chromosomal distribution of ‘missed’ and ‘extra’ gene changes. Showing the proportion of gene changes (from the copy number and LOH overlap analysis) identified as either ‘missed’ (white bars) or ‘extra’ (black bars) during the whole-genome amplification procedure and their chromosomal distribution (normalized to chromosome length). Graphs A–F show data from six different paired normal and dysplasia samples and compare the data generated using unamplfied (genomic) and amplified DNA. Proportion of changes is represented as a fraction with respect to all changes detected by a respective methodology.

In order to assess whether there were any patterns in the chromosomal location of the changes which were not detected, or were introduced by the amplification process, the genes identified as ‘missed’ or ‘extra’ were analysed according to their chromosomal start region normalized to chromosome length. This showed no consistent bias in location to particular chromosomal regions between different DNA samples (**Figure 1**).

### Functional and topological characterization

**Figure 2:**
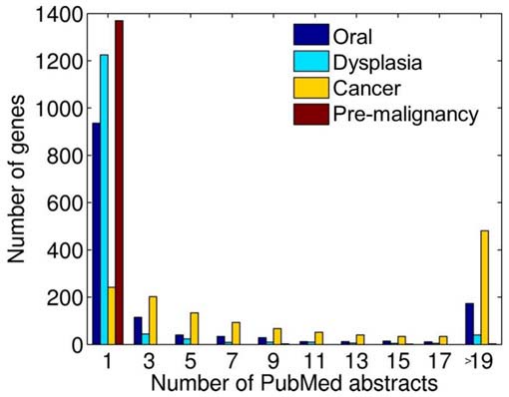
Automated PubMed search of 3112 genes with changes in oral dysplasia. Of the 3112 genes, 1375 (44%) had at least one PubMed cited abstract, suggesting the experimental data reflected acceptable coverage of current molecular knowledge in oral dysplasia and carcinogenesis.

A comparison of our findings with the current molecular knowledge of the genetics of oral carcinoma and premalignancy, was undertaken using an automated PubMed search of genes identified in both gDNA and amplified samples (‘experimentally identified genes’, *n* = 3112) using the keywords ‘oral’, ‘dysplasia’, ‘cancer’ and ‘pre-malignancy’. Of the 3112 experimentally identified genes, 1375 (44%) had at least one abstract per search term (**Figure 2**). Additionally, 1383 of these genes (44%) have known somatic mutations implicated in cancer, as curated by the Catalogue of Somatic Mutations in Cancer (COSMIC) database [11].

**Figure 3:**
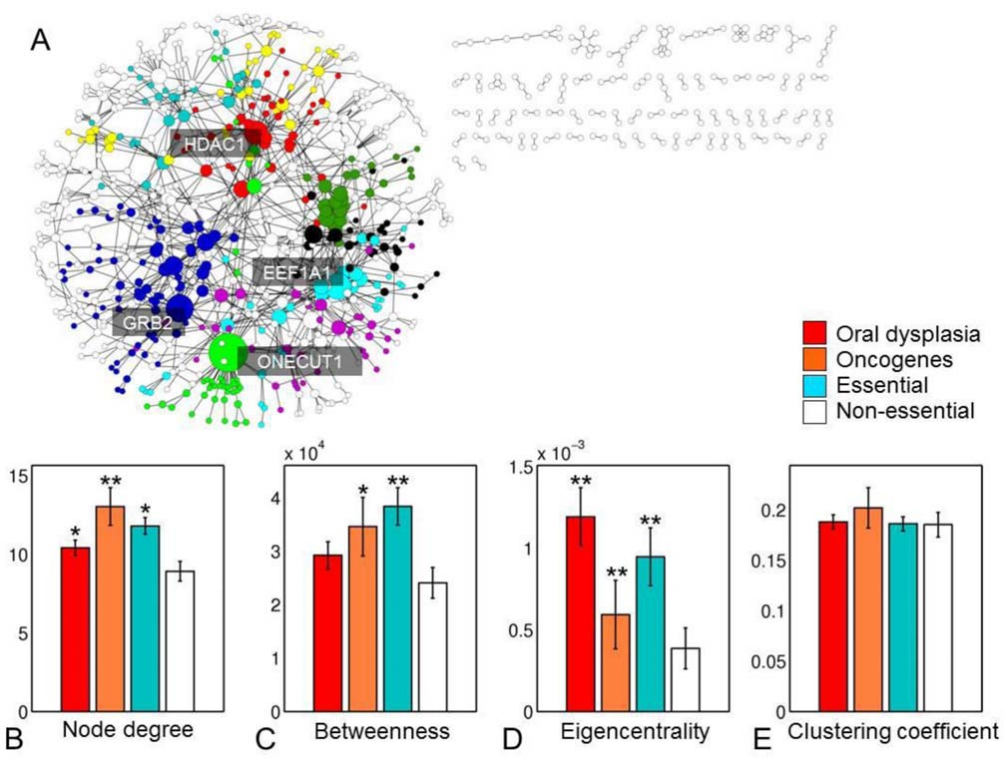
Topological analysis of oral dysplasia associated genes. **3A**) High confidence human protein-protein interaction (PPI) subnetwork consisting of 867 genes with changes in oral dysplasia and 1270 interactions. Node color corresponds to distinct gene communities identified by the Louvain method (see Methods). **3B–E**) Comparison of topological properties of oral genes (*n* = 1551), oncogenes (*n* = 171), essential genes (*n* = 1331), and non-essential genes (*n* = 413) in the PPI network. *p<0.05, **p<0.001 vs. non-essential. MEAN±SEM.

To present systems-wide properties of oral genes, experimentally identified genes were mapped onto the high-confidence human PPI network (see **Methods**). Interestingly, while 1551 (50%) of these genes could be mapped to the PPI network, 867 genes contained 1270 direct interactions with each other and formed an oral dysplasia sub-network. This sub-network was highly modular (modularity = 0.79), while the most highly connected genes included ONECUT1 (*n* = 43 interactions), GRB2 (*n* = 29 interactions), and HDAC1 (*n* = 24 interactions). For the 1551/3112 genes that could be mapped to interactome topological parameters, such as node degree, betweenness, eigencentrality, and clustering coefficients were calculated (**Figure 3B–E**). These topologies were compared to corresponding values for oncogenes (*n* = 171) as well as to essential (*n* = 1331) and non-essential (*n* = 413) genes (see **Methods**). The experimentally identified genes appeared to occupy significantly distinct topological locations compared to non-essential genes, as demonstrated by higher node degree (*p* = 0.02) and eigencentrality (*p* = 7.5×10^−5^) (**Figure 3B–D**). There were no significant differences in the respective betweenness, centralities and clustering coefficients. It is also important to note that these genes' topologies were significantly different (1.4×10−9≤*p*≤0.02) from oncogenes and essential genes.

## Discussion

The use of SNP genotyping platforms for copy number and LOH analyses with fragmented DNA has been well described [3], [4], [8], [12]. DNA from formalin-fixed tissues can be analysed provided stringent sample quality criteria are met, and the assay protocol and analysis are adapted accordingly [3], [10]. It must be possible to amplify the DNA to products of at least 300 bp [3], [10], [13], and hybridisation of the correct quantity of labelled PCR product to the chip is critical for copy number analysis. PCR performance with degraded DNA is less efficient and may require extra PCR reactions [3], [4], [10]. When analysing the data for copy number or LOH, a fragment length filter (based on the achieved PCR fragment length for each sample) is applied to reduce noise from SNPs located on large fragments that are inefficiently amplified [4], [10]. With these precautions in place, DNA from FFPE tissues can be analysed for copy number and LOH [3], [4], [12].

Despite this, the use of fixed clinical samples remains limited by physical sample size. Indeed, in this study eight of the available tissue samples failed to produce enough DNA for this reason. Amplification is one solution for analysis, and the genotyping of amplified DNA from good quality samples has previously been shown to correlate well with paired unamplified DNA [7], [8], [9], [14]. However, the subsequent performance for copy number and LOH analyses of suboptimal DNA remains contentious [15], [16] and the artefacts introduced by WGA of DNA from FFPE tissues are unclear. We aimed to determine whether amplified DNA from FFPE tissues could be used to generate meaningful data for genotyping, copy number, and LOH changes.

We have presented data from 10 micro-dissected DNA samples isolated from normal tissue and epithelial dysplasia, with a range of degrees of chromosomal instability. Part of each sample was amplified, and then both the unamplified gDNA and amplified DNA were hybridised to SNP 6.0 chips with appropriate precautions (Table S1). Filtering sample SNP data to exclude DNA fragments exceeding 350 bp left 131,429 SNPs for analysis. SNP fragment size is randomly distributed across the genome, so that loss in resolution does not compromise information from specific genomic regions [4], and only reduces the signal ‘noise’ associated with degraded samples [10].

Our internal method control call rate was 90.7% which is in line with other studies also using DNA from FFPE tissues in the analysis [3], [4]. This reduction in call rate, compared to studies only using good quality DNA, most likely reflects the effects of the inclusion of FFPE samples in the Birdseed (v2) RLMM genotyping learning algorithm. As expected, SNP call rate performance from unamplified gDNA and amplified DNA from FFPE tissues was lower than from unfragmented control DNA, but similar to that found in other studies using FFPE DNA and similar array platforms [3], [4]. Heterozygous and homozygous call rates were also slightly reduced in a manner consistent with the reduced call rates in FFPE samples (**Table 1**). There was no evidence to suggest a significant reduction in the proportion of heterozygous call rates caused by allelic dropout in either type of FFPE samples (gDNA or WGA DNA) during the DNA preparation and array procedures.

It was encouraging that, of those SNPs that failed to give a genotype call in the WGA samples, over half also failed to give a genotype call in the gDNA matched pair. Only homozygous AA calls showed a significant reduction (2.23%, p = 0.038) in no-call rates in the WGA samples (**Figure 1**), suggesting that there might be some allelic amplification bias towards AA calls in the amplification procedure. On average 76.5% of SNPs were called in both the matched gDNA and WGA DNA pairs. Of these, 82.4%±5.9 were concordant between the two samples (**Table 2**), lower than control DNA and its WGA pair (95.3%), but still correctly calling a large number of the genotypes.

Genotype concordance between unamplified gDNA and amplified DNA was lower than obtained from good quality DNA, for which up to 99% has been achieved [6], [7], [9], [14], [15]. Mead *et al.* achieved a similar concordance level with their degraded (not FFPE) DNA on early Affymetrix Nsp-Mendal arrays, but only by filtering the majority of SNPs out of the analysis and thus reducing the call rate of these samples to 24% [8]. This earlier study was limited to MDA amplification by the early Affymetrix platforms. It is to be expected that FFPE DNAs will not perform as well as control DNA, and the 82.4% concordance between the FFPE gDNA and its amplified paired DNA should be regarded as an excellent result.

Paired analysis was performed using a similarly amplified normal tissue DNA as a reference for the amplified dysplastic sample. The number of copy number changes detected was reduced in the WGA DNA samples compared to their matched gDNA pairs, but concordance (both samples detecting the same type of change) was high at 99.5%±0.4, indicating very few mismatch changes.

Changes that were ‘missed’ in the amplified pair (50.3%±22.1) were on average twice as likely to be amplifications as deletions, and changes ‘extra’ to the amplified pair (34.9%±27.7) twice as likely to be deletions as amplifications. These findings are interesting and complementary, suggesting that the whole-genome amplification procedure has failed to amplify some fragments efficiently, rather than introduced extra copies, most likely due to the reduced PCR-ability of fragmented DNA.

Our data suggest that around 50% of the changes in the original FFPE gDNA sample will be accurately detected (mean concordance 95.5%±0.4) in the amplified DNA, but that around 50% will be missed. They also suggest that approximately 35% of the changes in the amplified DNA will have been introduced.

Disappointingly, less than 30% of the LOH changes in the gDNA were detected in the amplified DNA. As there was no trend to reduced numbers of LOH regions detected in amplified pairs, more than 70% of the changes detected in these samples had been introduced as artefacts. These findings can be explained either by allelic dropout during WGA, or failure to genotype correctly due to increased noise, and the latter is more likely because there is no increase in homozygous calls in the WGA samples.

The Birdseed genotyping algorithm is a clustering algorithm [17] and requires training on 50 or more samples for greatest genotyping accuracy [18]. For this analysis, the 20 samples were supplemented with HapMap data, increasing the ‘noise in the system’. Therefore, our concordance for genotyping and LOH is the minimum achievable, and would improve with additional samples as the number of ‘missing’ and ‘extra’ changes is reduced.

The ability to overlap copy number and LOH data provides information about copy neutral LOH (acquired uniparental disomy). Of those genes detected as changed in both samples, 86.5%±8.9 were concordant (both samples detecting the same type of change e.g. amplification with or without LOH, deletion with or without LOH, or copy neutral LOH). Interestingly, this analysis showed the least ‘missed’ and ‘extra’ changes, but still over 45% of the changes in the gDNA sample were still not detected in the amplified DNA. There was no consistent pattern of chromosomal location of these ‘missed’ and ‘extra’ changes between DNA samples, suggesting that they result from random errors due to the degradation of the DNA, rather than the WGA procedure itself.

Automated PubMed searches for the 3112 genes that were identified by changes in both gDNA and amplified samples, confirms that our results offer a reasonable reflection of the published repertoire of genes with putative associations with oral dysplasia and carcinoma. Indeed, 44% of genes identified were already mentioned in the literature. Additionally, interrogation of the human PPI network and SNP data revealed that 867 genes (28%) with genomic changes are linked by 1270 physical protein-protein interactions, suggesting that these changes may be functionally relevant. Furthermore, dysplasia-associated genes occupied topologically central positions in the human interactome, implying that a genetic disruption in these genes is more likely to manifest itself in an abnormal phenotype [19], [20]. It was of interest to note that the oncogenes and essential genes had the highest node degree and betweenness centrality values overall, reinforcing their alleged detrimental role in disease [20]. These techniques further support the contention that the data obtained by amplification from FFPE samples are consistent with general topological principles of tumorigenesis [21] and have biological relevance, and this confirmatory data supports the validity of our methods.

In conclusion, our data support the research use of whole-genome amplification for DNA from FFPE tissues for SNP array analysis of genotype, copy number, and LOH; although inaccuracies preclude its use for diagnosis or treatment planning. Approximately 50% of the changes detected in the unamplified DNA will also be detected in the amplified DNA with a very high accuracy. WGA adds a layer of complexity and a consequent reduction in reliability. However, analysis remains meaningful, and concordance would improve as the number of samples increases. Error rates indicate the importance of an independent measure of the biological significance of the findings, for instance by functional bioinformatic comparison. Unlike frozen tissue samples, FFPE tissues are readily available from pathology archives, and may be the only type of sample available for research into rare diseases or longitudinal studies. The use of whole-genome amplification allows the exploitation of this valuable resource of genetic material for biomarker and functional genomics studies.

## Methods

### Ethics Statement

The study was approved by the Guy's Hospital Research Ethics Committee and the UK Patient Information Advisory Group, reference PIAG 4-09(f)2003.

### Tissue Samples and Laser Capture Microdissection (LCM)

Dysplastic oral epithelium and normal muscle were identified in 18 oral mucosal biopsy samples (FFPE), from six individuals, from the diagnostic pathology archive of the King's College London Department of Oral Pathology. Sections were cut at 12 µm thickness onto UV-treated 1 mm PALM membrane slides (Carl Zeiss Ltd, Welwyn Garden City, UK) and air dried. Slides were stained in preparation for LCM, after an overnight incubation at 50°C, using sterile equipment and DNAase-free solutions. The staining procedure was: removal of paraffin by two ×1 minute incubations in Xylene (Sigma-Aldrich, Dorset, UK), rehydration of the tissue by decreasing concentrations of ethanol (Sigma-Aldrich); two ×30 second incubations at 100%, a 30 second incubation at 95%, and 15 seconds in PCR grade water (Fisher Scientific, Loughborough, UK). The slides were then stained in Mayer's haematoxylin (VWR International, Lutterworth, UK) for 5 seconds, and washed in PCR grade water. Slides were dehydrated in graded ethanol, air dried and then stored at 4°C for up to one week. LCM of dysplastic epithelium, and normal underlying muscle, was performed using the PALM MicroBeam LCM microscope and stored at −20°C.

### DNA Extraction and Whole-Genome Amplification

DNA extraction was performed using the QIAamp DNA Micro kit (Qiagen Ltd, Crawley, UK). The only change to the standard protocol was to increase the Proteinase K digestion time to three days with daily additions of 10 µl of Proteinase K (Qiagen Ltd). Extracted DNA was stored at −20°C and DNA quantity was calculated using the Quant-iT Picogreen dsDNA kit (Invitrogen, Paisley, UK).

Amplification of the DNA was performed using GenomePlex WGA2 kit (Sigma-Aldrich). 100 ng of DNA was amplified and the resultant DNA was purified using the GenElute PCR clean-up kit (Sigma-Aldrich). DNA was stored at −20°C and DNA quantity was calculated as above.

### DNA Quality Control PCR

Primer sets were designed using Primer 3 for varying size amplicons of GAPDH DNA (NI_009759 ±2 Kb). Primer sequences are: for 300 bp product – ‘GACTCACCCTGCCCTCAATA’ and ‘CCCTGTAGCCTGGACCTGAT’, for 350 bp product – ‘CACACAGCTAGGGTGCAGAG’ and ‘TTCCAGGTCACCCTACAGGA’, and for 400 bp product – ‘AACCGGGAAGGAAATGAATG’ and ‘GGGAGCACAGGTAAGTGCAT’. The 25 µl PCR reaction included 5 ng DNA, in HotstartTaq PCR buffer with 2 mM MgCl_2_, 200 µM per dNTP, 0.2 µM of each primer, and 2.5 U of HotStartTaq (Qiagen). The PCR cycle was 95°C for 15 minutes, 45 cycles of 94°C for 30 seconds, 53.2°C for 30 seconds, and 72°C for 90 seconds, followed by a final 72°C incubation for 10 minutes. PCR products were visualised on a 2100 Bioanalyzer (Agilent Technologies, Stockport, UK) using DNA 1000 chips (Agilent Technologies).

### Affymetrix SNP 6.0 Array Protocol

No changes were made to the Affymetrix SNP 6.0 array protocol, other than starting with two aliquots of 500 ng DNA per sample, and performing at least double the number of Nsp and Sty PCR reactions (in the correct ratios) to achieve the required final pooled DNA quantity of 200 µg. This DNA was then fragmented, labelled and hybridised to the SNP 6.0 chip according to Affymetrix protocols. These changes to the standard protocol were made according to the Affymetrix Technical note for FFPE samples [10].

### Array Analysis

Hybridised chips were scanned and the resultant intensity ‘.cel’ files were imported into Affymetrix Genotyping console 2. The Birdseed (v2) RLMM algorithm was used to generate a genotype for each SNP, using the HapMap SNP 6.0 sample data set to increase the sample number past the 50 sample minimum threshold for this algorithm. Subsequent analysis was performed using the Partek Genomics Suite, filtering data by fragment length as described in the Affymetrix Technical Note [12] and by Jacobs *et al*, 2007 [4]. Genotype concordance was calculated as a percentage of the number of genotypes that were the same between two DNA samples, compared to the total number that were called in both.

Paired analysis (copy number, LOH, and overlap of the two) was performed with the Partek Genomics Suite, using matched normal DNA as a reference (Table S1). Gene lists were produced for each changed region and these were compared between the non-amplified and amplified samples. Concordance was calculated as a percentage of the concordant gene changes between the two pairs, compared to the total number of genes changed in both.

The distribution of ‘missed’ and ‘extra’ gene changes was measured by identifying the chromosomal start location of a changed gene (bases) and normalizing that position to the total number of bases in the respective chromosome. Because genes contained unequal number of changes, gene frequency was represented as a fraction of all changes in a sample.

### PPI network analysis

A high confidence human PPI network, comprising 57228 interactions among 11203 proteins, was assembled from yeast two-hybrid experiments, predicted interactions via orthology and co-citation, and curation of the literature [22]. The PPI network was represented as an undirected and unweighted graph where proteins correspond to nodes, and interactions between them correspond to edges. Gene-phenotype information was downloaded from the Mouse Genome Database (MGD) [23] and each mouse transcript was mapped to the respective human homologue using the Ensembl genome browser [24]. Oncogenes, essential, and non-essential genes sets were selected if allelic mutations in a single gene manifested in tumor, lethal, or absent phenotypes respectively. To evaluate node degree, betweenness, eigencentrality, and clustering coefficients associated with each gene set, individual genes were mapped to the human PPI network. Node degree is defined as the total number of interactions that connect to a given gene Betweenness is the measure of gene importance within the network, where genes that occur on many shortest paths between other genes have higher betweenness. Eigencentrality is a measure for how well connected a gene is to other highly connected genes in a network. Clustering coefficient is the degree to which genes tend to cluster together [25]. Statistical significance of differential topology values was measured using the Wilcoxon rank sum test. P-values≤0.05 were considered significant. The Louvain method for optimizing modularity was used to estimate the tendency of nodes in the PPI to form communities [25].

## Supporting Information

Figure S1 **Comparison of changes between Non-amplified gDNA and Amplified DNA.** Showing the average percentage of changes identified in amplified samples as a percentage of the non-amplified samples. Changes are represented as concordant (blue) and non-concordant (red) between the two sample types. Changes only identified in the non-amplified samples, therefore, ‘missed’ in the amplified samples are green, and only in the amplified samples, therefore ‘extra’ are purple.
(TIF)

Table S1 **Affymetrix SNP 6.0 Array Sample Information.** Showing the number of individual SNP 6.0 array datasets produced using DNA from formalin fixed paraffin embedded tissues and a blood internal method control. Matched paired gDNA and whole genome amplified DNA was used from 10 tissue samples (both normal and dysplastic tissues) taken from biopsies from four patients. Patients with multiple dysplastic epithelium samples were taken at least 6 months apart.
(DOCX)
