# Supplementary figures and images for: Copy Number and Loss of Heterozygosity Detected by SNP Array of Formalin-Fixed Tissues Using Whole-Genome Amplification
Source: PLoS One. 2011 Sep 26;6(9):e24503. doi: 10.1371/journal.pone.0024503 (PMC3180289; doi:10.1371/journal.pone.0024503)

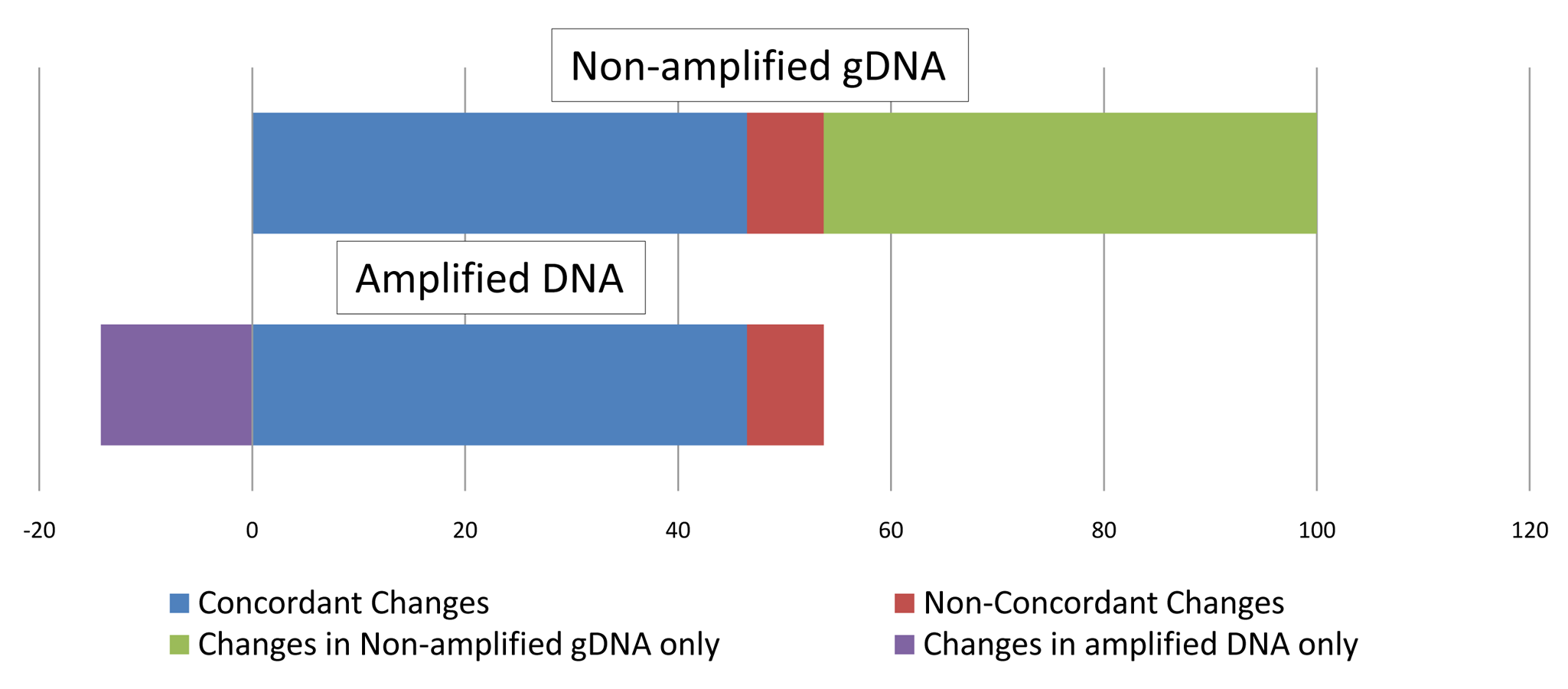

Supplement: Figure S1 — Comparison of changes between Non-amplified gDNA and Amplified DNA. Showing the average percentage of changes identified in amplified samples as a percentage of the non-amplified samples. Changes are represented as concordant (blue) and non-concordant (red) between the two sample types. Changes only identified in the non-amplified samples, therefore, ‘missed’ in the amplified samples are green, and only in the amplified samples, therefore ‘extra’ are purple. (TIF) [file pone.0024503.s001.tif]
