# Supplementary material for: Copy Number and Loss of Heterozygosity Detected by SNP Array of Formalin-Fixed Tissues Using Whole-Genome Amplification
Source: PLoS One. 2011 Sep 26;6(9):e24503. doi: 10.1371/journal.pone.0024503 (PMC3180289; doi:10.1371/journal.pone.0024503)
Supplement: Table S1 — Affymetrix SNP 6.0 Array Sample Information. Showing the number of individual SNP 6.0 array datasets produced using DNA from formalin fixed paraffin embedded tissues and a blood internal method control. Matched paired gDNA and whole genome amplified DNA was used from 10 tissue samples (both normal and dysplastic tissues) taken from biopsies from four patients. Patients with multiple dysplastic epithelium samples were taken at least 6 months apart. (DOCX) [file pone.0024503.s002.docx]

| **Patient** | **Sample Type** | **gDNA Extraction** | **Whole Genome Amplification of gDNA** |
| --- | --- | --- | --- |
| **1** | **Muscle** | Yes | No |
|  |  |  | Yes |
|  | **Dysplastic Epithelium** | Yes | No |
|  |  |  | Yes |
| **2** | **Muscle** | Yes | No |
|  |  |  | Yes |
|  | **Dysplastic Epithelium** | Yes | No |
|  |  |  | Yes |
| **3** | **Muscle** | Yes | No |
|  |  |  | Yes |
|  | **Dysplastic Epithelium 1** | Yes | No |
|  |  |  | Yes |
|  | **Dysplastic Epithelium 2** | Yes | No |
|  |  |  | Yes |
| **4** | **Muscle** | Yes | No |
|  |  |  | Yes |
|  | **Dysplastic Epithelium 1** | Yes | No |
|  |  |  | Yes |
|  | **Dysplastic Epithelium 2** | Yes | No |
|  |  |  | Yes |
| **Internal Method Control** | **Blood** | Yes | No |
|  |  |  | Yes |

**Supplementary Table 1. Affymetrix SNP 6.0 Array Sample Information.** Showing the number of individual SNP 6.0 array datasets produced using DNA from formalin fixed paraffin embedded tissues and a blood internal method control. Matched paired gDNA and whole genome amplified DNA was used from 10 tissue samples (both normal and dysplastic tissues) taken from biopsies from four patients. Patients with multiple dysplastic epithelium samples were taken at least 6 months apart.
